# FOODLIT-PRO: Food Literacy Domains, Influential Factors and Determinants—A Qualitative Study

**DOI:** 10.3390/nu12010088

**Published:** 2019-12-27

**Authors:** Raquel Rosas, Filipa Pimenta, Isabel Leal, Ralf Schwarzer

**Affiliations:** 1 WJCR-William James Center for Research, ISPA-Instituto Universitário, Rua Jardim do Tabaco, 34 1149-041 Lisboa, Portugal; filipa_pimenta@ispa.pt (F.P.); ileal@ispa.pt (I.L.); 2 Department of Psychology, Freie Universität Berlin, Habelschwerdter Allee 45, 14195 Berlin, Germany; ralf.schwarzer@fu-berlin.de; 3 Department of Clinical, Health, and Rehabilitation Psychology, SWPS University of Social Sciences and Humanities, 53-238 Wroclaw, Poland

**Keywords:** food literacy, qualitative, definition, influential factors, determinants

## Abstract

Poor eating habits are increasing the prevalence of weight-related issues, such as diabetes and cardiovascular diseases. Given the demand to improve individuals’ food knowledge and competencies aiming at healthier behaviours, the current investigation explores the concept of food literacy. Considering the lack of a shared understanding of food literacy, this study aims to explore food literacy’s domains, influential factors and determinants. Using a qualitative deductive-dominant content analysis, 30 experts from food-related fields were interviewed. The obtained outcomes were compared to available food literacy frameworks. Agreement among inter-raters was nearly perfect (k = 0.82). Yielding a total of 184 codes nested within 19 categories, identified domains were Origin, Safety, Choice and Decision, Select and Acquire, Plan, Preserve, Prepare, Cook, and Knowledge; influential factors included Nutrition, Psychological, Health, Learning Contexts, Policy, Industry, Sustainability, and Social and Cultural; External determinants were “Access to Food-Related Information”, “Perishable and/or Unreliable Food-Related Information”, “Family Dynamic and/or Identity”, and “Professionals’ Unpreparedness on Food-Related Expertise”, and Internal determinants included “Prioritise Food”, “Convenience and Practicality”, “Time and Financial Management”, “Previous Food-Related Habits”, and “Innate and Learned Flavour Preferences”. In conclusion, more than half of the identified attributes (62.5%) are corroborated by the current literature. However, the manifested content unmatched with the current frameworks of food literacy literature express food-literacy-related fields of action, knowledge, competencies, and determinants that have not yet been explored. As such, this study provides new and useful information concerning food literacy definition and development, by identifying its domains, factors of influence, and potential determinants. Moreover, this work paves the way for new measurements and interventions within this field.

## 1. Introduction

With rising rates of overweight over the last decades, the Food and Nutrition Action Plan 2015–2020 from the World Health Organization reports more than 50% of European adults with excessive body weight [1]. This call for action also highlights that poor eating habits are major risk factors directly linked to overweight issues and related to other noncommunicable diseases—such as cardiovascular diseases, diabetes, and some cancers. As such, healthy dietary habits are key not only to prevent and manage these diseases, but also to enhance quality of life [1]. With the eating patterns in developed countries poorly aligned with health-related recommendations, this increment of diet-related problems has been connected to the lack of both knowledge and skills on how to operate within a progressively intricate food system [2,3,4]. Given that most industrialised food environments are currently characterised by highly processed, ready-to-eat, low-cost foods with deficient nutritional value, individuals’ education and empowerment are needed to transform competencies and behaviours to navigate within these food systems towards healthier dietary choices [3,5].

In light of these issues, the current investigation explores the recently emerged concept of food literacy [3,6,7,8,9,10,11,12,13]. Despite being an increasingly recognised term, there is not yet a shared understanding of the construct’s meaning and its components, resulting in a lack of theoretical consensus [8,9]. Most acknowledged empirical conceptualisations of food literacy include (i) the framework of Vidgen and Gallegos from Australia [3,7,10,14,15,16,17,18,19,20], and (ii) the work of Desjardins and colleagues from Canada [21], which was later on revised by Thomas and coauthors [12,13,15]. Also from Canada, conceptualisations from (iii) Slater and coauthors [7,15,18] and from (iv) Cullen and colleagues [7,15,18,22,23] are considerably mentioned within the literature.

Overviewing these conceptualisations, (i) Vidgen and Gallegos [3] developed their framework through the qualitative analysis of both young adults’ and food experts’ perspectives (Delphi process with researchers and practitioners from education, community, and health sectors), describing food literacy as “the scaffolding that empowers individuals, households, communities or nations to protect diet quality through change and support dietary resilience” (p. 54). Thus, the authors created a model concerning food literacy “inter-related knowledge, skills and behaviours” (p. 54) within the four domains—Plan and Manage, Select, Prepare, and Eat—that represent and determine food intake. Similarly, the work of (ii) Desjardins and coauthors [21] started with a thematic analysis of perspectives from at-risk teens, young parents, and pregnant women to understand food literacy; from this qualitative study, a framework with two models (A and B) emerged—model A comprising personal dimensions of food literacy (food preparation skills and experience, organisational, food and nutritional knowledge, and psycho-social factors), and model B integrating external determinants of food literacy (social–cultural environment, food and facilities, living conditions, and learning environment). Also, the authors provided the definition of being food literate as “being knowledgeable and confident that one can regularly prepare meals that taste good and make one feel good, as well as having the organisational skills to find and use resources (human, financial, informational, material, time) to optimally make this happen” (p. 68). Subsequently, Perry, Thomas, and their colleagues [12] conducted a scoping review of the literature that led to the identification of five main themes—Food and Nutrition Knowledge, Food Skills, Self-Efficacy and Confidence, Ecologic, and Food Decisions. Later on, this team conducted a Delphi process with food-related professionals (researchers, practitioners, policy and decision makers within academia, community, health, and nongovernmental sectors) to build consensus on these attributes, resulting in a final framework with the same five main themes but rearranged content within these themes.

Likewise, (iii) Slater and coauthors [7] applied a Delphi technique with food and nutrition experts (dieticians, teachers, and nutrition/culinary students); however, this work intended to achieve consensus on food-literacy-related competencies specifically required by youth transitioning to an independent living through adulthood. As such, Slater and colleagues [7] developed a framework of food literacy competencies for young adults, which comprises three domains—(1) Functional Competencies: Confidence and Empowerment with Food; (2) Relational Competencies: Joy and Meaning through Food; and (3) Systems Competencies: Equity and Sustainability for Food Systems—that accentuate the diversity within food-literacy-related competencies. Differently, (iv) Cullen and colleagues’ “Food Literacy Framework for Action” [23] was solely driven by a scoping review of past literature. In this work, the authors propose a definition of food literacy as the conjuncture amidst community food security and individual food-related skills, highlighting the influence of one’s social, cultural, and environmental contexts in order to be food literate.

Most of these frameworks present multiple similarities concerning food literacy’s domains and definitions, as shown in the work of Truman, Lane and Elliot [24]; though not including the recent studies by (ii) Thomas and colleagues [13] and (iv) Slater [7], this scoping review identifies how food-related skills and behaviours, choices, knowledge, emotions, cultural and social aspects, and food systems are frequently identified as themes concerning the definition of food literacy. However, the lack of consistency among these frameworks—for example, the absence of cultural aspects within the frameworks of both (i) Vidgen and Gallegos [3] and (ii) Desjardins and coauthors [21], the lack of knowledge as a theme in (iv) Cullen’s framework [23], and the poorly explored theme of attitudes, motivation and other psychological and emotional factors within current literature—appears to be significant [24]. In fact, across contemporary literature, food/nutrition knowledge and practical skills/competencies are the most common aspects incorporated by food literacy definitions, with limited manifestation of surrounding contexts (such as social, cultural, political, and environmental contexts) as part of food literacy, despite their identification as influential aspects in order to be food literate [22,23].

Moreover, in addition to the majority of research originating from Canada and Australia, European studies focusing on food literacy are still scarce [24]. Northern and central European countries—specifically the United Kingdom, Italy, France, Netherlands, Switzerland, and Denmark—recently started to develop food-literacy-related research [24,25]. Particularly in Portugal, the ministry of health designated the National Program for the Promotion of Healthy Eating as one of the priority programs to be developed by governmental actions; this aims to improve the population’s nutritional status by encouraging the availability and consumption of foods that integrate a healthy eating regime [26]. Nonetheless, evidence-based research that provides an understanding of food literacy’s domains, determinants and influential factors, in order to contribute for the development of food-related knowledge, behaviours, skills and general food systems for the Portuguese population and context is yet to be conducted.

Therefore, this study integrates a project designated as FOODLIT-PRO: Food Literacy Project and aims to explore food literacy in terms of: (1) its domains, which can be understood as components that integrate food literacy; (2) its influential factors, perceived as contextual and surrounding spheres of action that can impact and be impacted by food literacy; and (3) its determinants, illustrating what can limit one’s food literacy.

To provide a clear perspective on the outcomes of this study concerning the current state of art, a comparison with current food literacy’s frameworks is presented along with these obtained results. Given the similarities between the current study’s methodological approaches and sample characteristics with frameworks (i) and (ii), considering the absence of the tailoring to a specific population like as characterised in the framework (iii) and the vulnerability of the framework (iv) by being uniquely based on a literature review, the obtained outcomes of this research are compared to the frameworks of (i) Vidgen and Gallegos [3] and (ii) the revised work of Thomas, Perry, and colleagues [13]. By doing so, it is intended to identify in which aspects the lack of consistency emerges and if new content arises, which may complement and expand previous ones within food-literacy-related current literature.

## 2. Materials and Methods

### 2.1. Study Design and Approach

In order to gather detailed data and to allow for a profound understanding of a particular concept or phenomenon of interest, qualitative methodology was chosen [27]. Particularly, this exploratory and cross-sectional study employed a deductive-dominant content analysis; this dominantly directed (both designations “deductive” and “directed” describe the use of previous findings for the analysis process) qualitative approach aims (i) to extend or corroborate an already existent theoretical framework that may benefit from further description, and/or (ii) to assess the (dis)similarity of a construct’s meaning in a distinct context [28]. Therefore, content analysis with a deductive-dominant approach was used to explore what defines food literacy and its domains, influential factors, and its determinants from the perspective of Portuguese experts from multiple food-related fields.

### 2.2. Recruitment and Sampling

Potential participants were recruited through direct contact (via email or telephone) with the institutions in which they were currently employed. Institutions were deliberately selected aiming to reach diverse organisations from across the (I) food-system and (II) other related fields—including not only (I, food system) food production, processing, distribution, marketing, consumption, and disposing, but also (II, related fields) policy-making, education, association with human and environmental health, and sustainability. Participant’s referencing (snow-ball sampling, nonprobabilistic convenience sampling) was also accepted as a recruitment strategy.

In total, 30 Portuguese experts (10 men and 20 women; Table 1) working in diverse food-related areas, with ages between 23 and 57 years (M = 38.4; SD = 8.60) from 26 different organisations—either governmental or nongovernmental as well as profit or nonprofit—were interviewed. Participants’ professions included nutritionist, public health doctor, researcher, director of politics’ development, psychologist, teacher, farmer, agronomist engineer, and cook, among others. The established contact with the participants took place between February and June 2018.

Inclusion criteria were (a) being 18 years or older, (b) minimum literacy and being able to answer to an extensive audio recorded interview, (c) working in fields (in)directly related with food, and (d) being responsible for feeding themselves. The criterion for (d) being responsible for one’s own feeding was evaluated through the minimum presence of one out of four possible items: holding responsibility over their food (i) choice and decision, (ii) selection and acquisition, (iii) preparation, and/or (iv) cooking (based on the model of Vidgen and Gallegos) [3].

The food-related fields in which the experts worked were grouped in five different ambits: education (E), encompassing basic education, high school, and environmental education; health (H), including nutrition, psychology, medicine; food policy (FP), with the establishment of food-related policies and development of national/international priority programs, among others; agricultural industry (AI), encompassing agricultural production and consulting; and commercial industry (CI), including marketing, brand management, product innovation.

### 2.3. Ethical Considerations

This study was approved by the Ethics Committee of ISPA-Instituto Universitário (ref. D/002/03/2018). Concerning the consent process prior to data collection, all participants were informed about the aim and methods of the study, as well as about the voluntary and confidential nature of their participation. The leading researcher assured all participants that their identities would not be revealed and provided, when requested, additional clarification concerning the study purpose. All participants provided oral and written consent for the interview, along with written authorisation for the audio recording. Confidentiality of the gathered data was guaranteed.

### 2.4. Data Collection

Prior to the interview, participants completed a socio-demographic (e.g., educational level, annual household income) and health behaviours (e.g., recent diseases, alcohol and tobacco consumption) questionnaire. Interviews were conducted both in the institutions that employed the participants and in the William James Center for Research (WJCR) facilities; in both contexts, a private setting with an individual room behind closed doors was provided. A total of 30 semistructured interviews were conducted by the first author in person (*n* = 21) and by telephone (*n* = 9) from March to June 2018. Interviews lasted from 25 to 120 min (M = 56.63) and were continued until data saturation was reached, meaning that no new contents were being added or explored. All interviews were audio recorded and fully transcribed; identifying information was edited by the time of the transcription. Randomised revisions were made to verify transcriptions’ accuracy.

### 2.5. Interview Protocol

The protocol for the semistructured qualitative interview was developed by the first author (a registered psychologist) and reviewed by this research team. The interview protocol used mostly open-ended questions, along with some leading and verifying questions; this allowed for further exploration of the subject and provided richness to the manifested content. The formulated questions were informed by food-literacy-related research published at the time of data collection, and aimed to explore food literacy’s domains, influential factors and potential determinants [3,13]. Further specific leading questions were formulated considering other food-related areas and influential factors, not yet thoroughly related to food literacy (e.g., food policy, and environmental concerns).

The protocol initiated with the following open-ended questions: (i) “What do you think is necessary for someone to eat according to one’s needs?”; (ii) “What do you understand by food literacy?”; and (iii) “In your opinion, what domains or components integrate food literacy?”. The current domains identified by previous research were explored subsequently, in form of leading questions—such as “What about knowledge (that is, facts and information gained through experience or education), what do you think people need to know to feed themselves according to their own needs?”, “What about skills (that is, practical techniques and skills), what do you think people have to know how to do to feed themselves according to their own needs?”, and “What about confidence and self-efficacy (that is, one’s belief about being efficacious), what do you think people have to master or feel in order to feed themselves according to their own needs?”, among others. A question concerning potential food literacy’s determinants—“In your opinion, which potential barriers or obstacles may arise when concerning people feeding themselves according to their own needs?”—was also inquired. Leading questions concerning topics not yet explored in-depth by current research were inserted with influencing field-related questions—“Which food policies do you think enable people to feed themselves according to their own needs?” and “Which environmental and sustainability-related aspects do you think endorse people to feed themselves according to their own needs?”.

This technique demonstrated to be the most suited concerning the protocol development in order to provide theme-related guidance to the participant, by starting with more general subjects and progressing to more specific content. Furthermore, the methodological approach chosen (deductive-dominant content analysis) affirms that targeted questions should follow general and flexible ones—which may help to identify initial coding categories [28].

### 2.6. Data Analysis

All interviews were analysed using the MAXQDA 2018 software [29], and the entire process of data analysis (including initial data analysis, secondary data analysis, and data verification) took place between April 2018 and January 2019.

The qualitative analysis process began with an initial reading of the transcriptions, occasionally making use of the correspondent audio record for further understanding. Given that previous theory of food literacy [3,13] informed the initial data analysis, some segments that appeared to be related with earlier theory were highlighted during the primary reading of the text. Therefore, the identification of those segments was possible through the use of predetermined codes derived from food literacy conceptual models, such as “planning and management”, “selection”, and “preparation” [3]; and “nutrition language”, “food techniques”, “food attitude”, and “sociocultural influences” [12,13]. These predetermined codes created a matrix of categories and correspondence codes, given its origin anchored in previous research. While developing the matrix, theoretical-based operational definitions of those categories and codes were accurately and objectively attributed. Despite the dominant deductive mode of reasoning applied in the beginning of the qualitative analysis—with existing theory informing the categorisation matrix—this was not the exclusive approach taken regarding the data analysis. When certain segments did not show a proper match to the codes and categories of the theory-informed matrix, new codes and categories inductively emerged [27].

After the development of the matrix and the coding process, all the data were reviewed independently by the leading researcher and a second member of this team. The constant comparison and reflection amid theory-informed and inductively-created codes and categories made along the coding process culminated in the development of a conceptual link between the existing theory and the added information from the present results.

### 2.7. Strategies for Trustworthiness

Given the importance of consistency in the coding process, the agreement among inter-raters is used as an indicator of the reliability among a given characterisation of a subject identified between researchers [30]. As such, to assess reliability, two researchers coded three transcribed interviews independently with the final coding matrix. The coefficient of Cohen’s kappa was used, because it is adapted for two coders and nominal categories [31,32], in this case the presence or absence of each code. Based on both researchers’ ratings, Cohen’s kappa indicated a nearly perfect agreement (k = 0.82). 

## 3. Results

The final qualitative analysis yielded a total of 184 codes, which were coded across 5981 text segments. For feasibility reasons concerning the results’ presentation, inclusion criteria of codes mentioned by a minimum of 10% of the sample (that is, three participants) was applied. Thus, a total of 3578 segments were coded by a revised total of 80 codes nested across 19 categories and their respective themes concerning food literacy domains, comprising nine categories; its influential factors, incorporating eight categories; and its determinants, integrating two categories.

The following display of the study findings includes the contrast against current literature reported by Vidgen and Gallegos (Appendix A Table A1) [3,25], Thomas and colleagues (Appendix A Table A2) [12,13], and Truman and Elliott (Appendix B) [17]. To provide a clearer reading of the results, the components of these theoretical frameworks were numbered, as done previously by Amouzandeh and colleagues [25]; the inventory of the frameworks’ categories and its respective attributes are detailed in the Appendices. 

### 3.1. Manifested Content and Sample Characteristics

Considering the diverse age range and to explore potential differences in the manifested content given by the participants, a Wilcoxon–Mann–Whitney nonparametric test was performed to compare the frequency of the manifested codes between participants below the age of 40 (Group 1) and those above the age of 39 (Group 2). Within the 184 manifested attributes, only the attribute “information seeking” belonging to the influential factor (iv) Learning Contexts, differed significantly between these two age groups (U = 57, W = 228, Z = −2.67, *p* = 0.03; mean rank Group 1 = 12.67; mean rank Group 2 = 19.75). This difference can be interpreted by the easy connection with diverse sources of knowledge (such as online platforms and social networks) that younger generations are more inclined to have nowadays, because these platforms may act as providers of constant notifications of information; thus, younger participants would not manifest the need to actively look for food-related information as much as older participants do.

### 3.2. Domains of Food Literacy

From the construct’s exploration, a total of nine categories emerged as identified domains of food literacy: (i) Origin; (ii) Safety; (iii) Choice and Decision; (iv) Select and Acquire; (v) Plan; (vi) Preserve; (vii) Prepare; (viii) Cook; and (ix) Knowledge. These domains incorporated a total of 22 codes, which are interpreted as the attributes that define the content of each of the food literacy’s domains (see at Table 2).

The majority of these domains and their attributes (meaning, categories and their respective codes) are sustained by current conceptual frameworks—such as the attributes of “cooking skills”, “matching ingredients”, and “using recipes” within the domain (viii) Cook, which are supported by both frameworks, and “planning skills” and “plan food intake ahead” from (v) Plan, being validated from one of the frameworks. More precisely, 11 (50%) out of the 22 emerged attributes are corroborated by both conceptual frameworks and seven (31.8%) attributes are supported by at least one theoretical model. However, four (18.2%) of the total attributes are not mentioned by these food literacy frameworks in any extent; for example, the attributes of “seasonality” within the domain (i) Origin, and “matching cooking techniques to ingredients’ nutritional value” from (viii) Cook. Particularly, and although neither of the frameworks against which these findings were compared refer “declarative” (also designated as critical) or “procedural” (also designated as functional) knowledge, these attributes from the domain (ix) Knowledge were considered in past food-literacy-related studies [23,24].

### 3.3. Influential Factors of Food Literacy

Regarding the analysis of food-literacy-related fields of influence, eight categories were identified as influential factors: (i) Nutrition; (ii) Psychological; (iii) Health; (iv) Learning Contexts; (v) Policy; (vi) Industry; (vii) Sustainability; and (viii) Social and Cultural. In total, 46 codes emanated from this analysis, defining the content and being interpreted as the influential factors’ attributes (see at Table 3). 

In contrast with food literacy’s domains, the majority of influential factors and their attributes are not yet explored by current research; this culminates in 23 (50%) out of the 46 attributes having no correspondence with the contrasting frameworks. Nonetheless, a total of 19 (41.3%) attributes manifest similar content with at least one of the conceptual frameworks; that is the case for attributes such as “tracking food intake” in (i) Nutrition, “critical analysis of food information” in (ii) Psychological, “information seeking” in (iv) Learning Contexts, “food: the glue of social connectedness” in (viii) Social and Cultural, among others. Specifically, within (ii) Psychological, the attribute of “sense of empowerment” is corroborated by the definition of the construct proposed by Vidgen and Gallegos [3]. Finally, only four (8.7%) of the attributes are supported by both existing frameworks: “awareness of food nutrients”, “awareness of nutritional needs”, and “interpret nutritional labels” from (i) Nutrition, as well as “food impacts health” from (iii) Health.

### 3.4. Determinants of Food Literacy

Concerning the exploration of food literacy’s determinants, two main categories were identified—(i) external determinants, comprising five attributes, and (ii) internal determinants, assembling seven attributes (see at Table 4). 

Differently from both domains and influential factors of food literacy, the manifested determinants were also compared with the framework of food literacy proficiency by Truman and Elliott [24] regarding factors than can limit behaviour change: knowledge, attitudes, skills/abilities, resources, and environmental conditions.

Of the identified 12 attributes belonging to both (i) External and (ii) Internal determinants, only one (8.3%) was corroborated by all frameworks [3,13,17]. A sum of three (25%) attributes were validated by at least two conceptual frameworks, and five (41.7%) were supported by a single conceptualization. Finally, three (25%) of these 12 attributes were not matched by any of the elements embraced by the three theoretical frameworks.

## 4. Discussion

This study aimed to identify qualitative content on three different outlooks concerning food literacy—domains within the construct’s definition, influential factors, and determinants—according to Portuguese experts from diverse food-related fields, and to compare these findings to the most recently developed empirical conceptualizations of food literacy. The match of 50 out of the total 80 manifested attributes (62.5%) with the compared frameworks (A, B, and C) demonstrates theoretical congruency among the obtained results with the current literature on food literacy, notwithstanding disparities across demographic, social, and cultural characteristics. However, the remaining 30 manifested attributes that have not found validation across the displayed frameworks demand further reflection.

### 4.1. Domains of Food Literacy

Greatly supported by the presented frameworks (18 out of 22 attributes; 81.8%), the manifested categories that designated domains of food literacy in this study—(i) Origin; (ii) Safety; (iii) Choice and Decision; (iv) Select and Acquire; (v) Plan; (vi) Preserve; (vii) Prepare; (viii) Cook; and (ix) Knowledge—are, mostly, already well-known in current research [3,13]. Despite the similarity of this arrangement of food literacy domains with the categories of framework A, essential differences require to be mentioned. First, the inclusion of domains that singularly concern food safety (Safety), food decision-making (Choice and Decision), food preservation (Preserve), and cooking skills (Cook) highlight the importance of these elements concerning one’s food knowledge, skills, and behaviours. Second, the addition of a domain strictly mentioning food-related theoretical and practical knowledge (Knowledge) paves the way for future diversity in outlooks for the quality of food literacy-related proficiency. Third, the dissolution of preparation (Prepare) and cooking (Cook) skills headlines that these two categories have distinct significance for one’s food literacy. Finally, it is essential to underline that, despite the similarity of these domains with current frameworks, the attributes that are incorporated within these domains outline aspects that were not yet stated with such detail (e.g., though being matched with framework B concerning the growing of food products within the attribute Food Systems”, the manifested attribute of “bio/organic: definition and impact” specifically addresses the need to better define and know the consequences for biological/organic food products).

However, the attributes of “seasonality” in (i) Origin, “matching cooking techniques to ingredients’ nutritional value” in (viii) Cook, and “declarative” and “procedural” (ix) Knowledge were not matched with the designated frameworks. In spite of this, the Food and Agriculture Organization (FAO) from the United Nations points out seasonality as essential to be considered to assess biodiverse food in dietary intake [33]. Moreover, seasonality has been mentioned as one of the aspects that defines balance among environmentally friendly and nutritionally beneficial eating patterns, being part of both the Mediterranean food wheel and food pyramid [34,35,36]. Regarding the alteration of foods’ nutritional value due to its cooking techniques, several recent studies have demonstrated not only that cooking methods can enhance the nutritional potential of diverse foods but also that nutritional losses may take place when preparing and cooking foods [37]. Having knowledge about how and why these changes occur demonstrates to be essential to the consumer because it will enable nutritional loss limit and improvement of foods’ nutritional value. Though considered in previous food literacy-related research [23,24,38,39], “declarative” and “procedural” knowledge were not stated in either frameworks A or B. As psychological constructs applied to food-related topics, declarative knowledge states the awareness of facts and processes regarding food sources, nutrition aspects, and other theoretical apprehensions; procedural knowledge refers to the set of practical competencies such as food-related decision making or food preparation skills—driven from the application of declarative knowledge previously stated [38,39]. The manifestation of (ix) Knowledge in this study restates the pertinence of structuring conceptual and practical food-related expertise within food literacy domains, because both can qualify different aspects of other identified domains; for example, one can know which foods are in season (“declarative” knowledge) but not shop for seasonal foods (“procedural” knowledge).

The obtained results and their confrontation with current literature intend not only to characterise food literacy’s definition and related domains within the Portuguese social and cultural contexts, but also to clarify and add up to the existent state of the art, expecting to contribute to further understand the meaning of being food literate.

### 4.2. Influential Factors of Food Literacy

In total, half of the attributes (23 out of 46 attributes; 50%) integrated within food literacy’s influential factors were corroborated by the highlighted frameworks. Though incorporating content similar to what is defined as food literacy in frameworks A and B, this study opted to display the influential factors separately from the domains of food literacy’s definition. Instead, the influential factors depict areas that actively interact with food literacy, which is a unique approach in current research. As such, this dissolution of the influential factors from food literacy domains express that these attributes—such as the awareness of food nutrients and one’s nutritional needs (Nutrition), the recognition of health-related consequences (Health), and the ability to seek food-related information in external environments (e.g., schools; Learning Contexts)—need to be understood not as being part of what food literacy means, but as aspects that interplay with what food literacy is, affecting and being affected by food-related skills, knowledge, and behaviours.

The attributes from the factors (i) Nutrition, (iii) Health, and (iv) Learning Contexts were fully matched with content from frameworks A and/or B. This is validated by the exhaustive research concerning food literacy-related aspects (knowledge, competencies, behaviours) within the fields of nutrition, global health, and learning environments, e.g., [40,41]. As such, the corroborated attributes integrating these influential factors mirror (i) nutrition-related skills (e.g., tracking food intake, interpreting labels, understanding specific language), (iii) health-related consequences (e.g., overweight and obesity), and (iv) learning mechanisms (e.g., information seeking, professional support). However, differently from the highlighted frameworks, this study comprehends these attributes not as part of the food literacy definition but as elements that influence and can be influenced by food literacy.

Though integrating mostly matched attributes, the factors (vii) Sustainability and (viii) Social and Cultural present aspects that require further reflection due to the lack of correspondence with both frameworks.

Belonging to (vii) Sustainability, the unmatched attributes were “food security: challenge of feeding the world”, “animal welfare” and “consequences of animal-origin foods”. Illustrating insufficient physical, social, and/or economical access to safe and nutritious food that allows for a healthy life, food insecurity is currently experienced by two billion people around the world [42]. Interconnecting poverty, economic growth, and nutrition, the 2030 Agenda for Sustainable Development highlights food security also as a priority in this call for action. Linked to food security, land use (which relates to the attribute “deforestation”), water pollution and greenhouse gas emission (related to other sustainability-related attributes, such as “impact of food importation” and “impact of the consumers’ demands”), and global human health, “animal welfare” and health are seen as part of a responsible and sustainable food system [43,44]. Taking into account the animal-source foods that are currently consumed across the planet, a safe supply of animal-origin food for people relies on animals’ health and their nutrition—demonstrating that the welfare of humans is closely connected with “animal welfare” [42].

Within the frame of sustainability, a mainly plant-based diet has been designated as the food regime that grants both health benefits and less environmental impact, considering the use of fewer natural resources in its production processes [45,46]. As “consequences of animal-origin foods”, a large environmental footprint has been pointed out: cropland use, greenhouse-gas emissions, and water use are aspects that make animal-source foods responsible for three-quarters of climate change effects [45,47]. Not only having significant environmental impacts, the entire food supply chain (from production to processing and retail) is acknowledged as affecting animal welfare and human health, general society, economy, and culture [45]. For diet-related sustainability purposes at a societal level, considering that some populations are entirely dependent on livestock, it is essential that diets are contemplated according to regional contexts [45]. Given the evidence that supports the connection between diet with both human health and environmental sustainability, and considering that further research on agrifood systems is recommended for food sustainability improvement, framing (vii) Sustainability as a factor of influence in a food literacy extensive conceptual structure appears to be relevant and well suited [45,48].

Referent to the (viii) Social and Cultural factor, the attribute “having a ‘healthy diet’ to be trendy” was the only one unmatched by both frameworks. With its potential role on social connectedness already recognised (related to the attribute “food: the glue of social connectedness”) [21], eating behaviours are closely connected with social attitudes and practices. Vast research identifies social factors such as social and mass media, market globalisation, and economic growth as drivers for individual and social behaviours, greatly influencing health and dietary-related practices [49,50]. This influence of the social context on diverse food-related aspects (such as food choices) can either be supportive of a healthful eating approach (linked to the attribute “social support”) or depict a barrier to the endorsement of a balanced food intake (related to the attribute “eating socially: gateway for unhealthier choices”). Consequently, social contexts and agents (such as schools, family and peers) are characterised as enablers of change in light of broader health tendencies [51]. As such, food-related trends (such as having healthful eating practices) may be learned and anchored within diverse social contexts; this would portray social aspects as main drivers to take these food behaviours into action, resulting in the acquisition of a “healthy diet” to be socially trendy. Thus, the influential factor (viii) Social and Cultural highlights these social and cultural surroundings that involve food-literacy-related behaviours and competencies on a daily basis.

Out of the eight categories identified as influential factors, three incorporated content mostly unmatched with the identified frameworks: (ii) Psychological, (v) Policy, and (vi) Industry. Identified as a psychological attribute, “creativity” has been indicated as an internal feature that enables an adequate food intake, especially when applied within the cooking domain [52]. Though unmatched with the designated frameworks, creativity was also stated by Desjardins and colleagues [21] in the first food literacy model developed by the Canadian team (later revised by Thomas and coauthors) [13], as part of psycho-social factors that integrate personal dimensions of food literacy. Also, part of the (ii) Psychological factor of influence, the attribute “critical analysis of food information” was supported by Thomas and colleagues’ framework [13] regarding the feature of nutrition literacy—defined as the ability to discriminate credible and inaccurate nutrition-related information, find this reliable information and use it for one’s benefit. Though not exactly with the same meaning, a critical analysis of food-related information incorporates the skill of identifying information’s reliability and validity, thus supporting this association. Furthermore, the attribute “sense of empowerment” was corroborated by Vidgen and Gallegos’ framework [3] concerning the definition of food literacy—described not only as a set of food-related knowledge, competencies, and behaviours essential for one’s feeding but also as the structure that allows for individual and societal empowerment aimed to conserve diet quality and supply for dietary resilience. Nonetheless, either understood as a goal or as a process, empowerment is a psychological construct often related with other psychological aspects, such as control, self-efficacy, or autonomy [53]. Hence, the attribute “sense of empowerment” was integrated within the influential factor that represents (ii) Psychological aspects that may impact or be impacted by food literacy.

Food literacy has also been stated as effective in impacting one’s behaviour by producing “health behaviour change” [9]. As an intricate process, health behaviour change involves multiple causal factors operating with diverse mechanisms where individuals navigate through distinct mindsets of behaviour change [54]. Though knowledge constitutes a precondition for this change, it is often not enough to transform individual behaviour (including food-related behaviour [55]; as such, the integration of this attribute as part of a factor of influence intends to acknowledge other psychological mechanisms needed to achieve health behaviour change related with food literacy. Considering that food is a primary indicator of both individual and group identity, a behaviour change technique with a potential to act as mediator for health behaviour change would focus on one’s food-related identity [51]. The attribute “identity associated with changed behavior” is a recognised technique that translates how one’s self-identification can be connected with the food-related behaviour that is aimed to change [56,57]; the affiliation of this attribute to a food literacy factor of influence aims to recognise the effect that self-identity can have on dietary behaviours. Still on the topic of behaviour change, the manifested attribute “habit formation” states how food-related behavioural patterns enact automatically, increasing the likelihood of the behaviours’ maintenance across time and unexpected circumstances [56,58]. Since food literacy concerns not only knowledge but also food-related behaviours, integrating attributes that focus on the acquisition and maintenance of these behaviours within the (ii) Psychological factor appears to be relevant.

The attributes “emotional eating” and “manage emotions to manage food intake” both refer to the influence of emotions on the context of food literacy. Expressing the tendency to overeat as a reaction to negative emotions, emotional eating focusses on how one’s emotional state impacts food-literacy-related behaviours [59]. Illustrating how decisions about food can be driven by emotions, “manage emotions to manage food intake” translates the need to have emotional management skills in order to manage one’s food intake [60,61].

Contrasting with emotions acting as driving forces for food-related behaviours, the attribute “positive sensorial and psychological consequences” underlines how food knowledge, competencies, and behaviours can induce sensorial and psychological outcomes. Already acknowledged by research in the field of food literacy, food (iii) Choice and Decision and eating behaviours are stated to interact with other domains of life and showed to be influenced by one’s preferences, such as taste or sensory perceptions [51,52]. Multiple studies have also recognised food literacy’s influence over not only physical but also psychological health and wellbeing [3,16]. These two attributes intend to represent that other outcomes not strictly related to nutrition, such as the sensorial pleasure of eating or the psychological contentment, can arise from dietary behaviours. Finally, the attributes “indulgence as part of the balance” and “emotionally nurture through food” emphasise other emotions that may also be related to food literacy. Since dietary behaviours frequently focus on strategies such as “tracking food intake” or “interpret nutritional labels” (attributes belonging to the factor Nutrition), an obsessive focus on food is often developed, leading to deprivation and subsequent periods of overindulgence [62]. Since overindulgence may cause feelings of guilt, developing a relationship with food that incorporates both healthy habits and the satisfied balance of food-related desires demonstrates a scenario in which the management of emotions benefits food behaviours [63]. Integrating “indulgence as part of the balance” of a healthy food intake within the (ii) Psychological factor portrays this need to balance both sides of food intake to be food literate.

Within indulgence, cooking is frequently associated with demonstrating affection to others or to take care of someone [64]. This act of demonstrating fondness to someone through food is represented in the attribute “emotionally nurture through food”.

Concerning its impact on the food and agricultural process—from food production to process, distribution, acquisition, consumption, protection, and disposal—the influential factor concerning (v) Policy aims to shed a light on the role of food-related public policy within the context of food literacy [65]. With the goal to frame consumer’s choices towards healthier directions, food-related policy approaches can be described as ‘soft’ policies, including providing information, education, and product labelling, whereas ‘hard’ policies encompass bans, fiscal measures, and mandatory regulations [66]. Therefore, the attributes “obligation to educate consumers” and “display products information” are examples of soft policies that influence food literacy. While the first attribute designates food policy’s role to educate the final consumer with information campaigns and educational actions, the second identifies food-related public policies’ function of guaranteeing transparency through the food chain aiming to promote a sustainable food consumption by informing the final client. Soft approaches as these are the most common actions within the domain of food and global health, having demonstrated positive impact and effectiveness [67,68]; however, these mostly preventive downstream actions demand personalised targeting (e.g., individual health education) ending up being successful mainly with smaller and target-specific populations. On the contrary, the attribute “regulation prior to consumption” is identified as a hard policy, because it targets food-market directly with mandatory regulations applied before the food decision from the final consumer; these approaches are characterised as more powerful, expeditious and cost-saving, considering that they are structural and upstream policies [66]. Regardless of having soft and/or hard approaches and to guarantee the greatest possible effectiveness, food-related policy-making must be developed and applied considering the target audience and its wider context [69]; the attribute “tailored interventions” mirrors this need to tailor the design and implementation of food-related interventions and policies. To proceed towards this, evidence also highlights the need to provide comprehensive strategies that include multisectoral policies (education, health, agriculture, etc.), allowing to tackle food-related issues with multi-stakeholder and multilevel approaches conceding systematic, interdisciplinary, and holistic food policies [70,71]—this approach for food policy is emphasised by the attribute “food in all policies: intersectoral policies”.

In the matter of mutual support and partnerships between (non)governmental agencies and major stakeholders, food industry has a unique role within food-related policies considering its close relationship with the final consumer. The integration of food (vi) Industry as an influential factor within this framework aims to translate the active role of the industry as a potential advocate for healthful eating patterns and an active agent in the implementation of food-related recommendations, cooperating in the achievement of favourable food-related outcomes. However, food marketing materialises a significant branch of this industry, linking a company with its consumer and aiming to influence food choices through food advertising [72]. Concerning sensory perceptions, evidence shows that intentional alterations of foods’ composition can modify the behavioural response to their consumption as a result of discrepancies in taste [73]. Regarding psychological variables, emotions are demonstrated to be increasingly used within advertisement due to their role in attitude formation, e.g., [74,75,76]. Thus, the attributes “flavour intensifiers for consumers’ loyalty” and “appeal to consumers’ emotions” express two different food industry strategies that are being used to impact one’s food choice.

Within mainstream strategies to impact consumers’ food-related habits, the attributes “marketing’s influence” and “social media’s influence” express two of the most frequently used avenues for food marketing disclosure: television and internet. Advertising on a large scale and with even short-term exposure being considered efficient, food marketing is highly controlled by sponsors and industry stakeholders, meaning that the provided information is not always for the benefit of public health [77,78]. Particularly with younger audience such as children and adolescents, cumulative exposure to food advertising through television and other social sources (e.g., social media platforms and sites such as Facebook, Instagram and Pinterest, food-related apps) is greatly associated with diverse food behaviours [79]. Due to this growing trend, policy makers are being increasingly solicited to take action in order to promote meaningful transformations in the food environment that may allow for more healthful marketing strategies. A law concerning the restriction of food advertising that appeals to the consumption of food and drinks high in calories, salt, sugars, and saturated fats, to minors below the age of 16 years is a recently implemented example of a hard policy within the Portuguese context [80]. Nevertheless, solid knowledge and competencies concerning food selection and acquisition, planning and management, and preparing and cooking food appear to be vital for one’s consumption of healthy foods [81].

Closing the influential factor of food (vi) Industry, the attribute “reaction to consumers’ demands” intends to emphasise that—despite the control that may be administered by public food-related policies or by industry stakeholders—consumers retain a powerful role within the food system, its marketing, and its final outcomes. With consumers’ interest expanding in the last two decades mainly due to the health and food-security-related crisis, demand for transparency within food-related information (concerning its origin, process, and production) has also increased. This demand for more information within the food chain leads to the judgement of food products concerning their quality, food safety, social consequences, and environmental impacts [82]. As such, industry’s transformation is perpetually possible since consumers’ demands act as a recognised driver of its performance—empowering consumers’ role in producing change within the food system.

Acknowledging the influential factors (ii) Psychological, (v) Policy, and (vi) Industry” as the most unmatched with the recent frameworks of food literacy emphasises not only the lack of participation from agents within these fields of action on food-literacy-related studies but also the scarce research that recognises these areas as being influential for individuals’ food literacy.

### 4.3. Determinants of Food Literacy

Most of the manifested attributes (9 out of 12 attributes; 75%) identified as determinants of food literacy were corroborated by at least one of the three highlighted frameworks (A, B, and C). Divided among (i) External and (ii) Internal, these determinants aim to indicate specific factors that may affect if and how individuals can develop and/or improve their food-related knowledge, competencies, and behaviours. Thus, within the (ii) Internal determinants, the unmatched attributes of “previous food-related habits”, “innate flavour preferences “and “learned flavour preferences” call for a more detailed analysis. 

Though concerning individual features, food choices and behaviours occur within a larger food environment that regards other characteristics. On this note, research is clear on how factors such as education, income, and political and sociocultural surroundings influence dietary habits and general health [83,84]. The attribute “previous food-related habits” emphasises how one’s familial and social contexts influence one’s food literacy, highlighting that habits formed earlier in life or within a specific family background may influence one’s lifelong habits [3]. As such, one’s “previous food-related habits” can impact if and how one’s behaviour is being changed. Particularly regarding food literacy frameworks, the initial frame developed by the Canadian team recognised the impact of familial, social, and cultural norms, values, and behaviours as Social–cultural Influences and Eating Practices [12].

Concerning one’s palate and sensory perceptions, the manifestation of the attributes “innate flavour preferences” and “learned flavour preferences” appears to be contradictory given the inherited and acquired enunciated nature of flavour. However, three fundamental senses of taste have been reported as related to flavour preferences: the taste for lipids (fats), the taste for sweets (sugars), and umami, referring to the taste of glutamate (savoury/salty flavours) [73]. Though it is possible for an innate preference to exist for either of these three senses of taste, studies indicate that most flavour preferences are learned [85,86]. More importantly, these attributes emphasise how flavour preferences can constitute personalised guidelines for one’s search of food knowledge, development and implementation of food competencies, and both intended and unaware food-related behaviours.

## 5. Conclusions

Food literacy research is still growing. Nevertheless, despite the increased investigation on this construct, congruency on its definition and measurement is yet lacking.

Within the major FOODLIT-PRO project, the present findings provide additional qualitative insights within the ambit of food literacy. Namely, this study presents in-depth information for the comprehension of the meaning of this construct, presenting a new arrangement of what comprises food literacy by identifying its domains. This research also supplies distinct understanding of the influential factors that play singular roles in the functioning and development of food literacy within a larger scope, by including hierarchical relations among diverse aspects integrating the food system. Finally, this study informs on which features can act to affect the development, growth, and enhancement of individuals’ food literacy, by identifying its determinants.

Integrating a sample with both men and women from food-related action fields as diverse as food policy, health, industry, and others, constitutes a strength, given that it provided for a wider comprehension of food literacy. Nonetheless, the absence of a Delphi structure to conduct this study could be regarded as a limitation. Although personal and contextual variables cannot be entirely controlled, conducting most of the interviews personally is a valued strength considering the analysis of nonverbal responses and the deepening of the responders’ answers, enabled by the interviewers’ presence. The inter-rater agreements as an indicator of reliability between researchers (k = 0.82) are also an important asset.

This study provides useful information on food literacy and contributes to a new understanding of the construct and its surrounding, hoping to assist in the advance of further measurement options and interventions’ protocol elaboration.

## Figures and Tables

**Table 1:** Participants’ socio-demographic characteristics.

Socio-Demographic Characteristics	Frequency (*n*)	Percentage (%)
**Affective-sexual relationship**		
Yes	27	90
No	3	10
**Educational level**		
Middle school	1	3.3
High school	2	6.7
Bachelor	19	63.3
Master	6	20
Doctorate	2	6.7
**Professional status**		
Active	29	96.7
Unemployed	1	3.3
**Professional Area**		
Education	7	23.3
Health	6	20
Agricultural Industry	5	16.7
Commercial Industry	8	26.7
Food Policy	4	13.3
**Annual Household Income**		
10,000 EUR or less	5	16.7
10,001–20,000 EUR	7	23.3
20,001–37,500 EUR	12	40
37,501–70,000 EUR	6	20

**Table 2:** Representation of the theme “Definition of Food Literacy”, its domains (categories) and respective attributes (codes), the coding against conceptual frameworks [3,13], an exemplifying quote and its respective author’s professional and demographic characteristics.

Definition of Food Literacy					
Category	Code	Frameworks		Example	Participant
		A	B		Area (Sex, Age)
Origin	Knowing Origin	2.2	1.1	“(…) to see this in an integrated perspective, like from the farm to the fork. From the primary production on how vegetables and fruits are produced, to animal production, to… everything, everything, everything, everything.”	CI (W, 38)
	Food Additives	2.2	1.1	“(…) about everything that is added to food (…) that is indispensable, for example dyes, preservatives, emulsifiers, etc.”	E (W, 36)
	How Origin Relates to Quality	2.3		“(…) to combine the agricultural production method to the final food’s quality (…)”	AI (M, 53)
	Seasonality			“(…) to have knowledge concerning the food’s season, (…) to buy seasonal products.”	E (W, 36)
	Bio/Organic: Definition and Impact		4.1	“(…) in the sense of what is biological food and what is biological food culture, biological production—it is not a massified production, it does not contemplate the use of pesticides. Then, of course, you will need a lot more space [for a biological production] than for a massified and systematical production. Which means that you will cut more trees to be able to plant your cabbages, to have your biological products…”	CI (W, 36)
Safety	Hygiene and Safety Practices	3.2	2.1	“(…) safe foods because, from a hygiene-and-food-safety point of view, there was no contamination. It is all right.”	E (W, 41)
	Pesticides and Herbicides		4.1	“(...) because there is a kind of information that I consider very important and which is not usually indicated in the label, nor will it come be so quickly… which is the level of pesticide residues, the presence of pesticides in the food.”	AI (M, 53)
Choice and Decision	Choice and Decision Skills	1.3; 2.1	5.1	“(…) the ability to make informed choices that ends up in reflecting in a better decision!”	FP (W, 28)
Select and Acquire	Selection and Acquisition Skills	2.1	3.2	“(...) when they go shopping, they must know what to bring home and what they should leave behind.”	E (W, 36)
	Nutritionally Equivalents Foods		1.2	“I think that what we find harder to learn is how we can replace (…) we must know that there are other ways of replacing a particular ingredient or product.”	H (W, 34)
Plan	Planning Skills	1.2		“This kind of (food-related) planning and organisation is important.”	AI (W, 43)
	Plan Food Intake Ahead	1.2		“This ends up being part of my weekend: having to spend hours, a couple of hours in the kitchen to make the rest of my snacks or my meals [for the following week].”	H (W, 28)
Preserve	Preservation Skills	2.2	2.1	“(...) and also conservation, (because) food can become tainted during the process between harvesting and consumption. The part of conservation is also important.“	AI (M, 53)
Prepare	Preparation Skills	3.1	2.1	“(...) you have to wash the food, you have to, I do not know, to peel off the skin or to do some kind of specific treatment.”	H (M, 39)
Cook	Cooking Skills	3.1	2.1	“Knowing how to cook—that’s another problem we’ve been watching! People cannot cook!”	FP (W, 28)
	Using Different Cooking Techniques	3.1	2.1	“(….) (ways of) cooking: whether it is steam, whether it is in the oven, whether it is boiled,... Whatever, it can be a competence.”	CI (W, 28)
	Matching Ingredients	3.1	2.1	“(...) basic notions of what should be the matching of foods, for example, (...) one has to know how to combine, know what ingredients should not be mixed with others.”	E (W, 57)
	Using Recipes	3.1	2.1	“(...) recipes books have everything standardised with measures, why is that? So that anyone is able to do that [recipe] and that it ends up, at least, similar to what is in the book, right?”	CI (M, 46)
	Matching Cooking Techniques to Ingredients’ Nutritional Value			“Methods to know how to adapt the (cooking) method to what you want to do, [and know] how to make the most of a food, right? Depending on the different cooking methods.”	E (W, 28)
	Cooking Motivation/Attitude		3.3; 3.4	“But there was really a... a boost in the interest of people in wanting to cook, to experiment, to go to the kitchen.”	CI (W, 44)
Knowledge	Declarative			“(…) is a set of skills, that one must have in order to be able to understand a certain concept.”	CI (W, 38)
	Procedural			“(…) it’s the knowledge that would get me to go from theory to action (...) I may know the theory but if I do not know how to apply it…”	FP (W, 28)

Professional areas: E—Education, H—Health, FP—Food Policy, AI—Agricultural Industry, CI—Commercial Industry.

**Table 3:** Representation of the theme “Influential Factors of Food Literacy”, its categories and respective attributes (codes), the coding against conceptual frameworks [3,13], an exemplifying quote and its respective author’s professional and demographic characteristics.

Influential Factors of Food Literacy					
Category	Code	Frameworks		Example	Participant
		A	B		Area (Sex, Age)
Nutrition	Awareness Food Nutrients	3.2	1.2	“(…) but people should be more aware of the (nutritional) value of food, I don’t want everyone to know the food wheel by heart… But people should have basic notions, at least.”	E (W, 57)
	Awareness Nutritional Needs	1.3; 4.1; 4.2	1.2	“First of all, it is necessary for people to know what their (nutritional) needs are— subject on which, I think, there is a great deal of ignorance.”	E (W, 41)
	Tracking Food Intake	4.2		“Ideally, we would like the consumer to do those (nutritional value) calculations that is, we are not talking about super precise by-the-book calculations, but general calculations. I think that is extremely important. ”	CI (W, 38)
	Interpret Nutritional Labels	2.2	3.1	“(…) and the concern to read labels is fundamental, (…) to understand what is written there, why it says what it says, to know what is written there, to know what that means.”	H (W, 36)
	Language		1.3	“For me, food literacy is what I was saying, (…) that is, disclose to people in simple, accessible language what those… well, let’s call them bad words on labels mean.”	CI (W, 41)
Psychological	Creativity			“(…) but we also need to do some creativity training.”	H (M, 39)
	Critical Analysis Food Information		3.1	“(…) and one has to have a critical analysis of things, but unfortunately lay people cannot. If for us—nutritionists—sometimes it’s hard, let alone for a lay person…”	H (W, 23)
	Sense of Empowerment	Construct Definition		(…) to make people capable, to give them the power to choose. One has to feel I am capable, meaning, one has to feel empowered, like I am capable of doing this. ”	H (M, 27)
	Health Behaviour Change			“(…) the psychology part, how these triggers are made… to trigger, through motivation, this behaviour change process.”	H (M, 27)
	Identity Associated with Changed Behaviour			“It can’t be an imposition because if people realise that they are not having their identity, (…) if we remove the identity from people, it will never work.”	H (M, 27)
	Habit Formation			“We create new habits, new routines, new ways of eating.”	CI (M, 40)
				“The skills and competencies we have to develop are linked to the automatisation of actions and thoughts, (…)”	H (M, 39)
	Emotional Eating			“(…) most people recognise that they eat other kind of foods when they feel frustrated or in a lower emotional state.”	AI (W, 43)
	Manage Emotions to Manage Food Intake			“(…) it is very much about the mental state, and people need to start managing their emotions first and only after to manage what is on the plate. This is so important.”	H (W, 23)
	Positive Sensorial and Psychological Consequences			“The food has to be good, it has to be tasty. People have to enjoy eating. We are programmed to enjoy eating.”	E (W, 41)
				“(…) I think we need to find (…) our own happiness to eat.”	H (W, 28)
	Indulgence as Part of the Balance			“(…) of course we all like to eat foods that are unhealthy and unnecessary to our diet, they are made for that… they are designed for that: to satisfy our taste buds, to satisfy our gluttony. (…) they must also exist (…) they will be the exception to the rule.”	E (W, 36)
	Emotionally Nurture Through Food			“(…) because it’s so important, when we make food for someone—in this case for our family—is a proof of love, isn’t it?”	CI (W, 38)
Health	Food Impacts Health	4.1	1.2; 4.1	“(…) not even medicine is as important to health as agriculture.”	AI (M, 53)
				“(…) obesity and overweight, because they don’t know how to eat.”	FP (W, 31)
Learning Contexts	Information Seeking		3.1	“What we can do as consumers, and I always come back to this, is to look for information. We have to search, to search a way to know, to seek knowledge.”	FP (M, 47)
	Schools		4.2	“Education in school should include training in this area, so that everyone could learn about this in public and mandatory schooling.”	AI (M, 53)
	Professional Support		4.2	“To have people receiving support, feedback, external reinforcement from health technicians (…) for implementation and initiation (in changing health behaviours) when it is not yet automatic—there has to be some external support.”	H (M, 39)
Policy	Obligation to Educate Consumers			“(…) an evidence-based national campaign that has to be carried out and it has to be led by public administration (…) a really consolidated campaign to increase food literacy—that is, to provide consumers with tools to allow them to understand these concepts,… which for us are simple but not so simple (for others), right?”	CI (W, 38)
	Display Products Information			“(…) ensuring that what is being made available [to the consumer] in consumption is informing enough. … If it is nutritional, it is nutritional. If it is about the price, it is about the price. If it is about the origin, it is about the origin. If it is about the way it was done, or about environmental issues, or about other concerns… whatever it is, it should be able to inform and, above all, not being able to deceive the consumer. This is our major concern. Transparent, clear, objective, valid and added-value information.”	FP (M, 47)
	Regulation Prior to Consumption			“I see the importance of these legislative measures as means for changing one’s habits, particularly at the palate level (…) I just focused on salt and sugar (…) there are certainties there (concerning salt and sugar) and, therefore, I consider that legislation is the best way to change the habits of society.”	H (M, 39)
	Tailored Interventions			“(…) the approach to be conducted needs to put the person at the center of the aim of the intervention and interaction. That is the paradigm that needs to exist.”	FP (M, 38)
	Food in All Policies: Intersectoral Policies			“The sustainability of those measures and interventions has to be taken care of and ensured by different policy approaches—meaning, health policies and agricultural policies must go hand-in-hand. A clear multisectoral approach—health, agriculture, industry, economics, everything.”	FP (M, 38)
Industry	Flavour Intensifiers for Consumers’ Loyalty			“Food additives that are used in the food industry aim to get the consumer to be flavour-anchored in that product.”	E (W, 41)
	Appeal to Consumers’ Emotions			“We sell empathy along with the products. I always give this example, wherever I am going to talk: products don’t sell themselves to you (…) for example, a famous soda brand sells happiness. It does not sell itself. And selling happiness is an emotional condition.”	FP (M, 38)
	Marketing’s Influence			“I think marketing helps a lot! (…) We turn on our TV and we are seeing food-related advertising at that time of the day. (…) When we arrive home, we never see advertising to healthy foods. Kids, on Saturday and Sunday mornings, while they watch cartoons on TV they are bombed with food-related marketing, only with foods that they should not eat on a daily basis.”	E (W, 36)
	Social Media’s Influence			“(…) one of the things we are seeing today is the phenomenon of social networks and digital influences. (…) What happens today is what is on the other side of the screens. Following these people (influencers), and some are good, really good influencers, because they are well informed and others not so much (…). People guide themselves by them, and that impacts a lot, meaning it has an impact on sales. It’s like advertising.”	CI (W, 31)
	Reaction to Consumer Demands			“The final consumer is the big driver (…). Within the value chain, the big driver of all this is the final consumer. It has no direct relation to the producer. (…) Current constrains on agri-food production are determined by consumers (…).”	FP (M, 47)
Sustainability	Food Security: Challenge of Feeding the World			“(…) and right now maybe we are taking much more than we are giving back, therefore when we are taking much more than what we are giving back we are not yet at the breakeven point. But it’s not easy to feed the millions of people that we are, and we (people in the world) keep increasing.”	CI (W, 41)
	Animal Welfare			“(…) if the cattle were freely in the pasture to be eaten, and there was no pressure for them to grow quickly to be slaughtered, there would be no such impacts.”	E (W, 41)
	Consequences of Animal-Origin Foods			“If we reduced the consumption of animals, we would have more plant-based foods: cereals, vegetables, fruits. And there wouldn’t be such a high impact on agriculture. And then there are other impacts of meat production: the amount of methane a cattle produces over its lifetime is too much for what the planet can handle. The amount of cattle we raise is the problem. The problem is not cattle, the problem is the amount of cattle that is quickly required for human consumption. Because cattle has been around for tens of thousands of years, and cattle wasn’t the problem.”	E (W, 41)
	Impact of the Consumers’ Demands		4.1	“There are consumption habits that are influencing agriculture and agri-food production a lot. So they are also beginning to interfere with the value and sustainability of the chain as a whole.”	FP (M, 47)
	Fair Trade		4.1	“(…) also, but not only, for example, to know if the place where we go to buy fruit supports its farmers.”	FP (W, 31)
	Local/National Trade		4.1	“(…) essentially local products, (…) this also had economic advantages for our national farmers, right? (…) and they are economically good for our economy.”	H (M, 39)
	Impact of Food Importation		4.1	“(…) instead of buying a mango that came from Brazil—if I consider the travelled distance in order to get to my plate, that it came by plane so it has consumed fuel and so it had its impact on environmental pollution—and choosing instead to consume something that is nationally produced and seasonal, I make a choice with a much smaller environmental impact.”	FP (W, 28)
	Seasonal: a Strategy for Sustainability		4.1	“If we have a sustainable food regime, then we have an adequate food regime. (…) because it’s regional, it’s local, because it’s seasonal. I think, currently, maybe people don’t have this notion or sensitivity. (…) Take the healthy and start implementing the sustainable. ”	H (W, 36)
	Deforestation		4.1	“Nowadays, the land that is used for agriculture is land that is stolen from the forests. And deforestation is a serious global problem as we know, isn’t it? We talk a lot about climate change and deforestation is one of the causes of climate change.”	E (W, 41)
	Single-use Food-related Items		4.1	“Start by reducing these kinds of things (…) the use of straws, (plastic) cups, plastics.”	AI (M, 52)
	Circular Economy		4.1	“(…) sustainable development goals, the circular economy. (…) we stop generating waste, because one thing is to waste, and another thing is not waste by continue to give it a second life. Until that residue has no chance at all, we will always keep trying to give it life.”	FP (M, 47)
Social and Cultural	Having a “Healthy Diet” to be Trendy			“I think that there is already a certain trend, at the societal level, of having a healthier diet. So, at the societal level… if our neighbor does one thing, we also start doing it. We are very much like that, we are very influential people (…).”	H (W, 28)
	Eating Socially: Gateway for Unhealthier Choices	4.3		“I mean, when my friends get together at someone’s house to eat, we’ll have pizza delivered (…). They will not be reading the food label; what matters is being reunited with friends. Food is just one way... It’s an aid mechanism... Food is always a second or third priority.”	E (W, 28)
	Social Support	4.3		“But obviously, in terms of social support, I would say… Having a group of friends or family who support us greatly in this kind of (food-related) changes and who don’t recognize it as a barrier to socialization.”	CI (W, 31)
	Food: the Glue of Social Connectedness	4.3		“(…) around the table and we know that people and families when they come together is around the table. It’s always…, there’s always food, there’s always food.”	H (W, 34)
	Evolution of Food Availability and Access		4.1	“(...) especially at the population level, years and years ago, citizens made healthy choices but they weren’t really choices, because people had no other option, right? Back then, little meat was eaten, meat was only eaten on Sundays or when someone was sick, and on day-to-day life the main resources were vegetables, cabbages, pulses, and bread.”	E (W, 41)

**Table 4:** Representation of the theme “Determinants of Food Literacy”, its categories and concerning attributes (codes), the coding against conceptual frameworks [3,13,24], an exemplifying quote and its respective author’s professional and demographic characteristics.

Determinants of Food Literacy						
Category	Code	Frameworks			Example	Participant
		A	B	C		Area (Sex, Age)
External	Access to Food-Related Information			1; 5	“I think the barrier is, above all, the communication, the access to information, as I just said… that seems to me, it seems to me to be key and important elements.”	FP (M, 38)
	Perishable and/or Unreliable Food-Related Information			1; 5	“(…) people also get a little lost because there is a lot of information and a lot of misinformation.”	CI (W, 38)
	Food Security: Lack of Food Access			5	“(…) when kids bring lunch boxes, there are some who don’t even… We also have kids who eat poorly because there’s lack of food at home (…).”	E (W, 57)
	Family Dynamic and/or Identity			5	“It’s about the example, and the child understands perfectly, isn’t it? I can’t be saying you have to eat the broccoli on your plate if I don’t have it on my plate, right? Automatically, she (the child) makes this comparison: if I don’t have it, then why does she need to eat it?”	FP (W, 28)
	Professionals’ Unpreparedness on Food-Related Expertise			5	“(…) in medical school, not even having a discipline about human nutrition… I mean, it gives the idea that medicine has nothing to do with food.”	AI (M, 53)
Internal	Prioritise Food	1.1		2	“(…) it’s about managing priorities, and everything in life is about managing priorities. (…) the consumer, what he should realize or what he should ideally realize is that his diet, the diet that is appropriate to his needs, his diet and that of his family is the number one priority! It should be first priority, first priority on the list, it should be first.”	CI (W, 38)
	Convenience and Practicality	1.1		2; 4	“(…) is accommodation, isn’t it? It is much more practical and convenient to take a box of precooked food out of the freezer and put it in the microwave than to exercise your imagination and start cooking your own food (…).”	E (W, 57)
	Time Management	1.1		4	“I think that management is the most important thing, if we can manage our time to go to the supermarket… and if not, to shop online (…). Our lives today don’t allow us as much (time) as we would like, so we go back to the issue of time management, right?”	CI (W, 41)
	Financial Management	1.1; 1.3	4.2	4	“(…) in the families we work with, the perception that they want to do better and different but they cannot, due to financial issues—this is the first! Comparing with their financial and economic situation, the family tries to make the best possible management of it. (…) For families with no income, it is easier to go to the supermarket to buy precooked foods; water, electricity, or gas are not spent in the same proportion when comparing to cooking a meal from scratch.”	E (W, 37)
	Previous Food-Related Habits				“(…) population niches where eating habits and eating behaviours are established practices, right? It’s about not knowing how to do it differently, because they never saw that a different reality could exist.”	E (W, 37)
	Innate Flavour Preferences				“I think it’s born with us, right? Feeling good and feeling that a specific food gives us pleasure or not. Therefore, we are born perhaps with a particular palate. It’s already there, isn’t it? Already there, our taste buds and what we like… So, it is born with us.”	CI (W, 41)
	Learned Flavour Preferences				“(…) in terms of flavour, it will taste much better (to eat) something with sugar than something with no sugar at all, right? Now the palate, the taste, is an educated thing. We can go training, experimenting and checking. (…) [taste] has to be educated, doesn’t it?”	CI (W, 41)

Professional areas: E—Education, H—Health, FP—Food Policy, AI—Agricultural Industry, CI—Commercial Industry.

## Appendix A

**Table A1:** Framework A [3].

Categories	Attributes
1. Plan and Management	1.1 Prioritise money and time for food
	1.2 Plan food intake (formally and informally) so that food can be regularly accessed through some source, irrespective of changes in circumstances or environment
	1.3 Make feasible food decisions which balance food needs (e.g., nutrition, taste, hunger) with available resources (e.g., time, money, skills, equipment)
2. Select	2.1 Access food through multiple sources and know the advantages and disadvantages of these
	2.2 Determine what is in a food product, where it came from, how to store it and use it
	2.3 Judge the quality of food
3. Prepare	3.1 Make a good tasting meal from whatever food is available. This includes being able to prepare commonly available foods, efficiently use common pieces of kitchen equipment and having a sufficient repertoire of skills to adapt recipes (written or unwritten) to experiment with food and ingredients
	3.2 Apply basic principles of safe food hygiene and handling
4. Eat	4.1 Understand food has an impact on personal wellbeing
	4.2 Demonstrate self-awareness of the need to personally balance food intake. This includes knowing foods to include for good health, foods to restrict for good health, and appropriate portion size and frequency
	4.3 Join in and eat in a social way

**Table A2:** Framework B [13].

Categories	Attributes
1. Food and Nutrition Knowledge	1.1 Food knowledge
	1.2 Nutrition knowledge
	1.3 Food and nutrition language
2. Food Skills	2.1 Food skills
3. Self-Efficacy and Confidence	3.1 Nutrition literacy
	3.2 Food and nutrition self-efficacy
	3.3 Cooking self-efficacy
	3.4 Food attitude
4. Ecologic Factors	4.1 Food systems
	4.2 Social determinants of health
5. Food Decisions	5.1 Dietary behaviour

## Appendix B. Framework C [24]

1. Knowledge (lack of information)
2. Attitudes (lack of interest)
3. Skills/Abilities (lack of acquisition/application skills)
4. Resources (lack of time)
5. Environmental conditions (context-specific limitations of home, school, food-choice environment, and social norms)
